# Editorial

**Published:** 1844-05-04

**Authors:** 


### THE MEDICAL EXAMINER.

### PHILADELPHIA, MAY 4, 1844.

In our last number we noticed somewhat in detail the 15th Annual Report of the Inspectors of the Eastern Penitentiary of Pennsylvania, with particular reference to the influence of solitary confinement on the health of the prisoners; since then we have learned some additional facts from Dr. Hartshorne, tending to confirm the position that such a system of imprisonment, when judiciously carried out, is by no means productive of the evils which many humane minds have apprehended.

The converse of this has recently been urged with great earnestness by the British press, and the opposite side as strenuously defended by Mr. Dendy and others. In a late number of the London Lancet, Mr. Dendy expresses his opinion that writers on the other side "are looking through a false medium, and prejudge the question from prejudice and a morbid sympathy with criminals. At all events, their observations are drawn forth solely by isolated and extreme cases, and the objections therefore to the solitary system become a question merely of degree."

This is certainly verified in the experience of our Penitentiary.

By a note from the Physician (Dr. Hartshorne,) dated on the 16th ult. we are informed that for six months prior to that date there had not been a single death in the prison: "and but one,—a case of introduced fatal disease,—has terminated since August, 1843," (eight months.)

We are by no means to be understood as undervaluing the importance to health of free exercise in the open air, but certainly so small an amount of mortality as this statement exhibits in so large a number of convicts, (nearly 500,) is evidence enough to quiet the fears of legislators and philanthropists as to the influence of that system of confinement on health, compared with the other systems of punishment elsewhere pursued.

---

In our first number of the present volume, (Jan. 13, 1844,) we spoke of the large number of Students in attendance at the University of Pennsylvania and Jefferson Medical College of Philadelphia. The Editor of the Medico-Chirugical Review, in the last number of that Journal, (April, 1844,) in some remarks on the condition of the British Metropolitan and Provincial Schools, quotes our observations, and finds therein occasion for the following illiberal expressions, viz:

"We cannot but remark with satisfaction the fortunate condition of the University of *Pennsylvania*. The 'drab-coloured men' may well make doctors of their sons, seeing that they can pay the matriculation fee with other people's money. We only trust, that that very fee is not *repudiated*, and that in that very respectable state, there is, in their dealings with each other, honour among thieves."

This sounds very like an attempt to imitate the smartness of the Rev. Sidney Smith. It certainly partakes much more of the temper of that *pious* divine, than of justice and propriety. The editor may, for aught we know, have suffered loss like him, by reposing too much confidence in the mercantile regularity of our political institutions; if so, we are sorry for it. But does it follow that the people of Pennsylvania, or the medical profession in the United States, are *thieves and robbers*, because bad or incompetent men have gained a temporary control of the fiscal affairs of the state? Does the history of England afford no parallel to the mortifying condition in which Pennsylvania is now placed? We regret this unfortunate departure from the dignified and manly tone generally maintained in that respectable publication, because of its unfairness, and because it encourages by example others who have less character and good sense to restrain them.

---

On another page it will be seen that the Clinical Lectures of Professor Dunglison, delivered at the Philadelphia Hospital, are concluded for the present season. They were attended from the beginning by an unusually large class of Medical Students, and Physicians, who have on repeated occasions testified their high appreciation of the instruction afforded them during the course by himself and colleague, and the abundant means for clinical observation, supplied by the wards of that extensive Institution. The list of subjects presented in the present report, beside those embraced in the surgical course of Professor Pancoast, and those also which fell under the notice of the Professors of the University of Pennsylvania, will afford to persons at a distance some idea of the character and extent of that great Hospital as a school of practical medicine.

To the gentlemen who have so kindly and so well reported these lectures, the Editor is greatly indebted.

Concurrently with the winter courses in the colleges, the lectures will be resumed at the Hospital by the same gentlemen by whom they have been heretofore delivered, and will be promptly reported in the Examiner. During the summer, regular reports of lectures delivered at the same place, at the Clinic of the Jefferson Medical College, and other public institutions in Philadelphia and the European Cities, will form a portion of the contents of the future numbers of this Journal.

